# Targeted lipidomics showed altered serum phosphatidylcholine levels in fearful dogs

**DOI:** 10.1371/journal.pone.0356063

**Published:** 2026-09-02

**Authors:** Luigi Sacchettino, Michele Costanzo, Sabrina Bianco, Iolanda Veneruso, Valeria D’Argenio, Viviana Orsola Giuliano, Pietro Lombardi, Francesco Napolitano, Danila d’Angelo

**Affiliations:** 1 Department of Veterinary Medicine and Animal Production, University of Naples Federico II, Naples, Italy; 2 Department of Molecular Medicine and Medical Biotechnology, University of Naples Federico II, Naples, Italy; 3 CEINGE-Biotecnologie Avanzate Franco Salvatore, Naples, Italy; 4 Department of Experimental and Clinical Medicine, University of Catanzaro Magna Græcia, Viale Europa, Catanzaro, Italy; 5 Department of Human Sciences and Quality of Life Promotion, San Raffaele Open University, Rome, Italy; University of California Riverside, UNITED STATES OF AMERICA

## Abstract

Fearfulness accounts for most disorders affecting family dogs, who generally experience several behavioral traits, including trembling, hiding, flinching at noises, panting, or ears held back. Intense and persistent exposure to threatening situations might escalate to phobia, which easily makes patients more prone to inter- and intra-specific aggression. Factors like pain, adverse environment, and poor nutrition, as well as medical conditions, may contribute to the onset and severity of fear, although molecular pathways underlying such a dysfunctional emotional status are not yet fully understood. Among them, growing evidence highlighted the importance of lipid dynamics in mood-related disorders, both in humans and animals. Accordingly, lipids are the main components of brain structure, facilitating neuronal activities, including energy storage, signaling, and membrane integrity. In the present work, we identified a distinct lipidomic pattern significantly altered in the serum of fearful dogs, characterized by the differential abundance of several lipid species, primarily phosphatidylcholines. This predominance of phosphatidylcholine dysregulation underscores the central role of membrane phospholipid metabolism in canine fear behavior and suggests that lipid dysmetabolism is associated with behavioral disorders in companion animals. Collectively, our data pave the way for a deeper characterization of the impact of lipid dysregulation upon mood-related dysfunctions in dogs and for identifying diagnostic biomarkers in veterinary practice to cope with this behavioral disorder.

## Introduction

Like humans, companion dogs can exhibit maladaptive behavioral patterns reflecting negative affective states, particularly fear and anxiety [1,2]. When persistent and functionally impaired, these patterns progress to clinical disorders, with generalized fear/anxiety representing a prevalent presentation in family dogs [3,4]. Affected animals exhibit behavioral and physiological indicators, such as hypervigilance, avoidance behaviors, and autonomic activation, that constitute the foundation for standardized clinical evaluation [5,6]. At present, behavioral diagnosis depends on behavioral observation, standardized assessments, and anamnestic history questionnaires provided by pet owners [7]. While clinically valuable, these methods remain inherently subjective, with studies demonstrating that behavioral interpretation significantly varies between experienced or inexperienced observers [8] and owner reports, that may be biased by individual interpretation and observational context [3,8]. Therefore, behavioral signs alone may not differentiate among various physiological states or predict individual treatment response, in addition to subjectivity, which has thereby prompted the search for objective and quantifiable biomarkers. The discovery of specific molecular fingerprints may reveal a better explanation of the animal’s affective state, promoting an evidence-based approach in behavioral veterinary medicine. Therefore, these indicators may improve clinical assessment, help to monitor the efficacy of treatment, and identify biological subgroups within complex behavioral phenotypes. Lipids are a major group of biomolecules involved in physiological processes, acting as energy-storing enzyme cofactors, emulsifying agents, vitamins, hormones, intercellular messengers, and structural components of cell membranes [9]. Alterations in lipid metabolism have been implicated in the pathogenesis of several pathologies, ranging from metabolic to neuropsychiatric disorders and anxiety. A growing body of research across species connects alterations in lipid metabolism to behavioral abnormalities [10–13]. In humans, phosphatidylcholine (PC) dysregulation has emerged as a promising signature of affective disorders [14,15]. While non-targeted metabolomics has identified metabolic alterations in fearful dogs [13], targeted phospholipid profiling in canine generalized fear remains unexplored. Circulating PCs may serve as peripheral lipidomic signatures, reflecting systemic metabolic dynamics and altered pathways related to central affective states within the gut-microbiota-brain axis [16]. Consequently, analysing circulating lipidomic profiles during routine clinical screenings could provide veterinarians with objective and quantifiable biomarkers to assess behavioral conditions and ensure animal welfare. Therefore, in this preliminary study, we investigated the serum lipidome in fearful and healthy family dogs to better understand the correlation between lipid profiles and generalized fear in dogs.

## Methods

### Ethical statements

All experimental protocols were approved by the Scientific Ethic Committee for Animal Experimentation (Reference number: PG/2023/0011527), in accordance with the Italian legislative Decree (N. 26/ 2014). The blood sample was taken during routine visits, as a part of screening for health problems. Written informed consent was obtained from each owner for the publication.

### Animals, clinical diagnostic criteria and inclusion parameters

The cohort of dogs described in this study was part of a broader research project investigating the bidirectional communication of the gut-microbiota-brain axis in the canine species [16]. Dogs in the fearful group were treatment naive. Inclusion required that dogs have no prior history of behavioral therapy or administration of psychotropic medications, including anxiolytics, antidepressants, sedatives, or nutritional supplements. The absence of pharmacological or behavioral intervention was verified throughout the animal’s life, to ensure that lipidomic profiles reflected the intrinsic physiological condition associated with fear behavior. Each dog underwent a comprehensive behavioral and clinical evaluation conducted by board-certified veterinary behaviorists at recruitment, approximately two weeks prior to blood collection, to guarantee that physiological data represented a stable emotional phenotype rather than a temporary state of excitement. The diagnostic method adhered to a multi-dimensional framework consistent with recognized principles of veterinary psychiatry and behavioral medicine [17]. Each evaluation lasted 60–90 minutes. The protocol began with an in-depth clinical history exploring behavioral ontogeny, environmental background, and emotional stability. This was followed by direct observation in a controlled clinical setting to monitor spontaneous interactions and responses to mild environmental challenges, including neutral social approaches or novel stimuli. A comprehensive physical examination was conducted to assess autonomic parameters and rule out concurrent medical diseases that could mimic or intensify behavioral symptoms [18,19]. To qualify for the fearful group, dogs were required to demonstrate functional impairment, characterized by a chronic state of distress that significantly compromised quality of life and adaptive capacity [17], alongside at least three behavioral indicators of negative affect: continuous hypervigilance with relentless environmental monitoring; persistent avoidance or escape behaviors; displacement behaviors, including contextually inappropriate lip-licking or yawning; and altered social signaling, such as social withdrawal or ambivalent proximity-seeking behaviors. In contrast, control dogs were selected based on a history of behavioral health and the display of calm, exploratory behavior throughout the assessment, meeting none of the inclusion criteria for the fearful group.

### Study population and sampling procedures

Fearful (case) and normo-behavioural (control) dogs (n = 8) were between one and eight years old (Table 1). The enrolled subjects, lived in different Italian cities, namely Caserta, Naples, Salerno (Campania Region), and Formia (Lazio Region). They had not received antibiotics in the four weeks prior to the collection of stool and blood samples, nor had they been given glucocorticoids, proton pump inhibitors and/or probiotics, herbal remedies, or dietary supplements. To minimize nutritional bias, all dogs were maintained on an overlapping diet consisting of high-quality commercial dry food. The nutritional profiles were comparable across the cohort, with a composition of crude protein (24–28% of total content), fat (11–18%), fiber (2.2–14%), ash (5.8–11%), and moisture (8–9%). All dogs were subjected to physical examinations by a licensed veterinarian, including recording their body weight and body condition score [using a 9-point scale: underweight (1–3), ideal (4–5), and overweight (6–9)]. Subsequently, they underwent a complete blood test, which also included the evaluation of thyroid function, coprological examination and protein electrophoresis, to rule out any co-morbidities, potentially related to behavioural issues [18,19]. A small patch of hair was shaved from the dog’s neck and topical anaesthesia [Eutectic Mixture of Local Anaesthetics (EMLA™) cream] was applied to the area before collection of a 5 ml blood sample from the jugular vein. Blood samples were collected from each animal between 7:00 and 10:00 am, immediately frozen on dry ice, and stored at −80°C until their further processing. Blood sample was collected during routine veterinary examinations, and as part of canine health screening; therefore, this study was performed without any additional suffering or discomfort to the animals. All the laboratory parameters that were evaluated in the dogs fell within the reference ranges. Chi-square test was carried out using GraphPad Prism 10 software.

**Table 1:** Demographics of dogs enrolled in the study.

ID/ Breed	Group	Sex	Years	Reproductive status	Kg	BCS (1–9 scale)	City (Region)
01/ Mongrel	Case	M	2	Neutered	18	4	Caserta (Campania)
02/ Pinscher mix-breed	Control	F	3	Spayed	5	5	Caserta (Campania)
03/ Weimaraner	Control	M	4	Intact	38	5	Salerno (Campania)
04/ Mongrel	Case	F	2	Spayed	14	4	Salerno (Campania)
05/ Mongrel	Case	F	5	Spayed	6	4	Salerno (Campania)
06/ Rottweiler	Control	M	2.5	Intact	50	5	Salerno (Campania)
07/ Mongrel	Case	F	1.5	Spayed	30	4	Formia (Lazio)
08/ Mongrel	Case	F	7	Spayed	7	5	Formia (Lazio)
09/ Mongrel	Case	F	2	Spayed	20	5	Formia (Lazio)
10/ Mongrel	Case	F	3	Spayed	15	4	Salerno (Campania)
11/ Weimaraner	Control	F	7	Spayed	30	5	Salerno (Campania)
12/ Mongrel	Case	F	4	Spayed	19	4	Salerno (Campania)
13/ Shepard mix-breed	Control	M	7	Intact	37	5	Naples (Campania)
14/ Hunting mix-breed	Control	M	5	Neutered	28	4	Naples (Campania)
15/ Mongrel	Control	M	6	Intact	22	4	Naples (Campania)
16/ Bobtail	Control	M	7	Neutered	50	5	Naples (Campania)

### Serum lipidomics analysis

The lipidome of fearful and healthy dog serum was characterized using a targeted metabolomics platform [20,21]. Lipid extraction, sample preparation, and mass spectrometry analysis were carried out according to the MxP Quant 500 protocols provided by Biocrates Life Sciences (Innsbruck, Austria) [22]. The MS-based lipidomic platform allowed the detection/quantification of 523 lipid species by flow injection analysis-tandem mass spectrometry (FIA–MS/MS), whereas the features with a high percentage (> 50%) of missing values or non-valid values were discarded in the post-analysis. The lipid classes included: acylcarnitines (AC, n = 40), cholesterol esters (CE, n = 22), glycerolipids, including diacylglycerols (DG, n = 44) and triacylglycerols (TG, n = 242); glycerophospholipids, including lysophosphatidylcholine (lysoPC, n = 14), acyl-acyl-phosphatidylcholine (PC aa, n = 38), and alkyl-acyl-phosphatidylcholine (PC ae, n = 38); sphingolipids, including sphingomyelins (SM, n = 15), ceramides (Cer, n = 28), and dihydroceramides (Cer, n = 8); glycosylceramides, including hexosylceramides (HexCer, n = 19), dihexosylceramides (Hex2Cer, n = 9), trihexosylceramides (Hex3Cer, n = 6). For each analyzed sample, 10 µL of serum were loaded onto a 96-well extraction plate, including positions for blanks, PBS, calibrants, and quality controls (QC). Samples were dried under nitrogen stream, derivatized with 50 μL of 5% phenyl isothiocyanate at room temperature, and then dried again. Metabolite extraction was performed by shaking samples with 300 µL of 5 mM ammonium acetate in methanol (30 min, 450 rpm), followed by elution through centrifugation (2 min, 2000 rpm). A 10 μL aliquot of the metabolite extract containing lipids was diluted with 490 μL of FIA solvent (provided by Biocrates) and mixed by shaking (10 min, 600 rpm). The prepared sample plate was subsequently analyzed by FIA-MS/MS using a Triple Quad 5500 + QTRAP Ready mass spectrometer (AB Sciex, Framingham, MA, USA) coupled to a 1260 Infinity II HPLC system (Agilent, Santa Clara, CA, USA). In detail, 20 μL was injected into the system within the mobile phase (FIA solvent) at a flow rate of 0.03 mL/min until 1.6 min, followed by flow rates of 0.20 mL/min for 1.6 min and 0.02 mL/min for 0.20 min. The electrospray ionization (ESI) source worked in positive ionization mode and the following parameters were used: curtain gas (CUR) 30 psi, collision gas (CAD) 8 psi, ion spray voltage (IS) 5500 V, temperature 450 °C, ion source gas 1 (GS1) 20, GS2 40. Targeted analysis of lipid species was performed by multiple reaction monitoring (MRM) in technical triplicates to control the analytical variability. Lipid quantification was carried out based on isotopically labelled internal standards and kit-specific calibration procedures by using the MetIDQ Oxygen software (Biocrates Life Sciences AG) [23,24]. The final dataset was composed of concentration values (μM) for 523 lipids targeted by MRM. “NaN” (Not a Number) values were reported for features with missing values or bad quantification.

### Lipidome data analysis

The lipidomic dataset was analyzed with the MetaboAnalyst 6 and GraphPad Prism 10 tools for chemometric and statistical analyses [25–27]. The technical triplicates of each sample were kept as individual features for most of the analyses reported. The dataset was imported into MetaboAnalyst, and data were filtered, normalized, log10-transformed and auto-scaled. The normalized dataset generated partial least squares-discriminant analysis (PLS-DA) models that were employed as supervised methods to detect group variance. Samples were classified according to different biological variables for data analysis. For sex-based analysis, animals were divided into males (n = 7) and females (n = 9). For age-based analysis, dogs were grouped into a young cohort (1.5–4 years, n = 9) and an old cohort (5–7 years, n = 7). Finally, according to fear status, samples were classified into case (n = 8) and control (n = 8). Replicates of the main groups (case and control) that deviated significantly from the main data cluster upon PLS-DA were recognized as outliers and excluded from the dataset. PLS-DA model performance and robustness were evaluated by the 5-fold cross-validation method using five components for the classification. To estimate the potential risk of overfitting, diverse parameters were used to assess model reliability: the accuracy, the goodness of fit (R²) and the predictive ability (Q^2^) of each model generated [28]. In addition, the PLS-DA model for the case animals was employed to predict the class of relevant features according to the Variable importance in Projection (VIP) score. Lipids with VIP score > 2.0 were selected as predictive of the analyzed phenotype. Hierarchical clustering analysis by heatmap visualization was obtained according to lipid abundance and the 30 most significant features by PLS-DA VIP were reported. Univariate statistics was performed using the normalized dataset by volcano plot analysis to select differentially abundant lipids. The fold-change was indicated as Difference, that is the difference between the mean (log)levels of one lipid in fearful dogs and controls. The significance threshold was set at p < 0.01 (–log10 p-value > 2). Parametric Welch’s t-test or non-parametric Mann-Whitney t-test were used as statistical tests according to the normality of the distributions, assessed by D’Agostino & Pearson test, to further validate the difference of significant lipids obtained from volcano plot analysis. For such tests, raw µM concentrations were used. Metabolite Set Enrichment Analysis (MSEA) was employed to investigate sublocalization of selected lipids. In addition, receiver operating characteristic (ROC) analysis was performed to evaluate the discriminatory power of the identified lipid features and their potential utility as biomarkers [29]. A biomarker model was generated using a PLS-DA algorithm for feature ranking. Lipid variables were ranked according to their importance in the model using the first latent variable (LV1), which reflects their contribution to class separation. The ROC curves were then generated using the subset of statistically significant lipids identified in the univariate analysis (t-test). Model performance was evaluated by calculating the area under the ROC curve (AUC), sensitivity, and specificity. Lipidomics data have been deposited to the EMBL-EBI MetaboLights database [30] with the identifier MTBLS11669.

## Results

### Fearful dogs exhibit increased phosphatidylcholine levels

**Fig 1:**
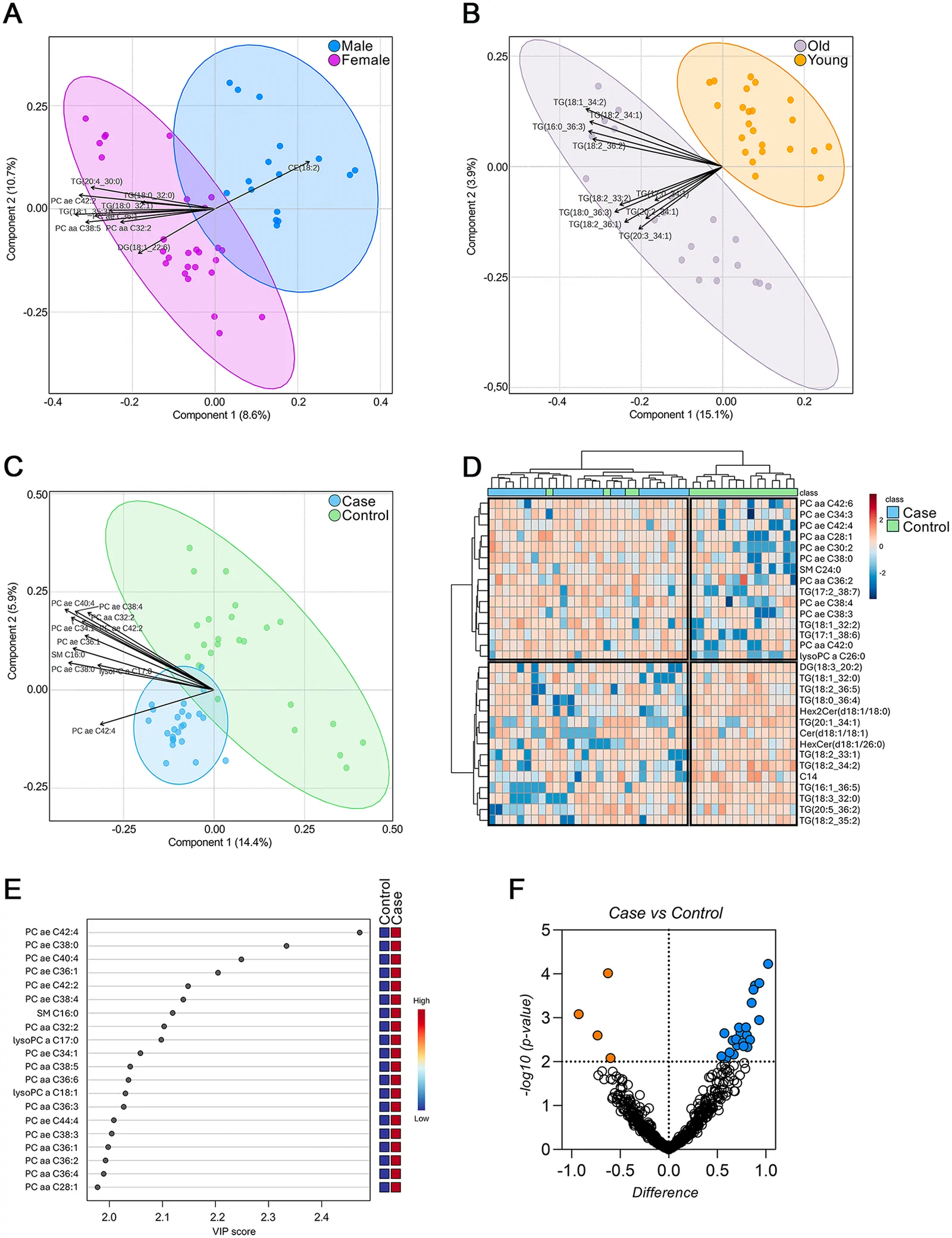
Serum lipidomics analysis and lipid changes in fearful (case) and healthy (control) dogs. Supervised multivariate analysis performed using Partial Least Squares-Discriminant Analysis (PLS-DA) on the lipidomic dataset to evaluate potential sources of variance. Score plots show the degree of groups variance and their relative loadings according to sample grouping based on (A) sex (male *vs* female), (B) age (young *vs* old), and (C) fear status (case *vs* control). (D) Heatmap showing groups and replicates clustering based on normalized lipid abundance. Black boxes comprise lipids with specific trends. (E) The 20 most significant VIP metabolites extracted from the PLS-DA model from case *vs* control comparison were reported. (F) Volcano plot analysis of significantly different lipids in the case *vs* control comparison. The blue and orange dots represent the significant increased and decreased lipids, respectively, in the case group. Non-colored dots refer to all the molecules identified in the dataset whose relative abundance is not significantly different between groups..

**Fig 2:**
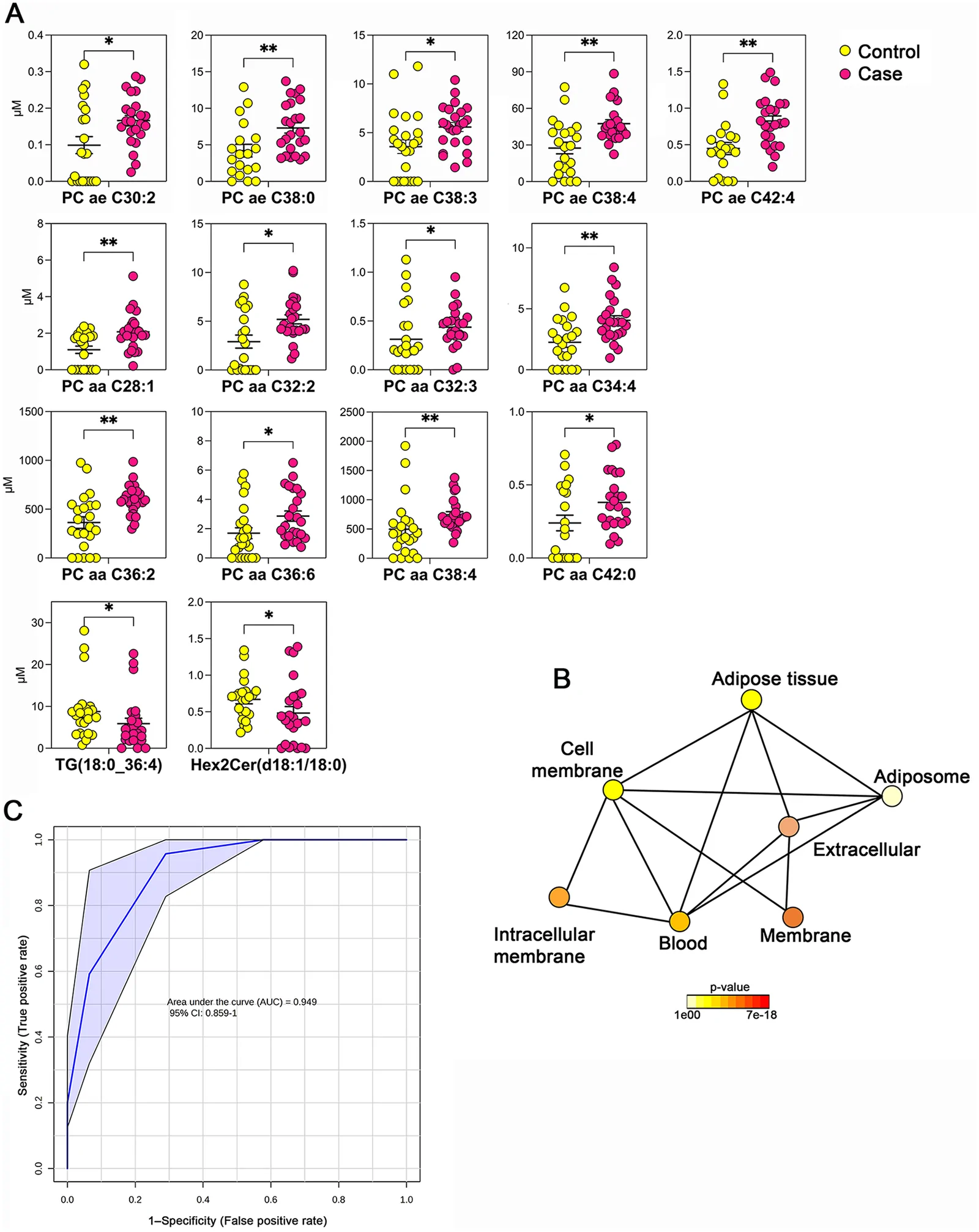
Functional and statistical validation of the selected lipid features. (A) Lipid levels were compared by unpaired parametric or non-parametric t-test according to the normality of the distributions; and significant lipids were reported with * p < 0.05, ** p < 0.01. (B) Metabolite Set Enrichment Analysis (MSEA) of lipid sublocalization using organ and tissue databases; color ranges are indicative of the p-value of the enrichment. (C) Biomarker evaluation of selected differential lipids in fearful dog. The ROC curve was generated using a PLS-DA algorithm, with AUC values calculated from the combination of the 15 lipids reported in (A).

Lipid changes in serum of fearful dogs were assessed by FIA-MS/MS using a targeted lipidomic approach. Overall, 1.7% of missing values were detected and imputed, whereas one lipid feature (free carnitine, C0) was discarded, as non-valid values were present in more than the 50% of the samples. After filtering and normalization, 522 lipids were correctly quantified, and the metabolomics profiles of study groups were analyzed by multivariate and univariate statistics. To ensure that the observed differences between fearful and control dogs were specifically attributable to the fear status rather than potential confounding factors, PLS-DA models were constructed by pooling all samples (regardless of their fear state) and evaluating the independent effects of sex and age as classification variables. When sex was used as a classification variable, no clear separation between groups was observed (Fig 1A) and the model showed low predictive ability (Q^2^ < 0.2), indicating that sex does not significantly contribute to the variance in the lipidomic profiles. In contrast, age-based classification resulted in a partial separation of samples (Fig 1B), although with moderate predictive performance (Q^2^ > 0.2), suggesting a discrete influence of age. The variables contributing most to this age-related variance were predominantly triglycerides (TGs). Notably, when comparing fearful (case) and healthy (control) dogs, the PLS-DA model showed clear separation between the two groups (Fig 1C), supported by good model performance metrics (accuracy = 0.8, R^2^ = 1 and Q^2^ > 0.4). The corresponding biplot highlighted distinct clustering of samples, with phosphatidylcholines (PCs) emerging as the primary contributors to group discrimination. Also, the hierarchical clustering performed by heatmap analysis showed a distinct distribution of the features in the two groups highlighting the 30 most significant lipids by PLS-DA VIP (Fig 1D). Subsequently, we extracted the VIP molecules from the PLS-DA model able to discriminate between case and control groups; of these, most lipids were represented by PCs and 17 species showed VIP > 2.0 (Fig 1E). Univariate statistics by volcano plot analysis identified the differentially abundant lipids in the binary comparison case *vs* control (Fig 1F). Specifically, 26 lipids were found significant, including 4 down-regulated and 22 up-regulated lipids in the case group. Of interest, the up-regulated molecules are almost represented by PCs species. Table 2 reports the significant lipids in the order from the less abundant to the most abundant with details of statistics, including P-value, –log10(P-value), Difference, standard error (SE) of difference, and regulation trend. The variation of the 26 differentially abundant lipids identified by volcano plot analysis was further validated through pairwise statistical comparisons using the non-transformed concentration values obtained from the MS-based lipidomic quantification. The comparison between case and control groups confirmed the differential regulation of 15 out of 26 lipids, comprising 13 phosphatidylcholine species, five PC ae (PC ae C30:2, PC ae C38:0, PC ae C38:3, PC ae C38:4, PC ae C42:4) and eight PC aa (PC aa C28:1, PC aa C32:2, PC aa C32:3, PC aa C34:4, PC aa C36:2, PC aa C36:6, PC aa C38:4, PC aa C42:0), plus one ceramide (Hex2Cer(d18:1/18:0)) and one triglyceride (TG(18:0_36:4)) (Fig 2A). To gain further biological insight into the origin and potential functional relevance of these lipids, Metabolite Set Enrichment Analysis (MSEA) was performed using organ-, tissue-, and subcellular-localization databases. This analysis indicated that the majority of the selected lipid species were associated with membrane structures and circulating lipid pools in blood (Fig 2B). Finally, the panel of 15 validated lipid features was used to construct biomarker models using a PLS-DA-based algorithm. Model performance was evaluated through receiver operating characteristic (ROC) curve analysis, which yielded an area under the curve (AUC) of 0.949 (Fig 2C), indicating a high discriminatory ability between fearful and control dogs. Overall, these results suggest that this lipid signature may represent a highly informative biomarker panel associated with fearful behavior in dogs.

**Table 2:** Significant lipids found differentially abundant in fearful dogs *versus* controls.

Lipid	P-value	–log10(P-value)	Difference	SE of difference	Regulation
Hex2Cer(d18:1/18:0)	0.00083	3.081	−0.9267	0.2591	Down
TG(18:0_36:4)	0.002523	2.598	−0.7332	0.2294	Down
TG(18:2_34:2)	0.000096	4.017	−0.6258	0.1465	Down
TG(18:1_32:0)	0.008309	2.08	−0.6002	0.2176	Down
PC aa C38:4	0.007551	2.122	0.542	0.1939	Up
PC aa C36:2	0.002239	2.65	0.5729	0.1769	Up
lysoPC a C28:1	0.008388	2.076	0.5927	0.2152	Up
SM (OH) C14:1	0.006154	2.211	0.6281	0.2187	Up
PC aa C34:4	0.003282	2.484	0.6571	0.2118	Up
PC aa C36:6	0.006936	2.159	0.6701	0.2371	Up
PC aa C36:5	0.003092	2.51	0.694	0.2222	Up
PC aa C32:3	0.004249	2.372	0.7029	0.2336	Up
PC aa C42:0	0.001678	2.775	0.7232	0.2167	Up
lysoPC a C26:0	0.002157	2.666	0.7395	0.2275	Up
PC ae C32:2	0.003678	2.434	0.766	0.2503	Up
SM C24:0	0.004445	2.352	0.7702	0.2574	Up
PC ae C38:0	0.001656	2.781	0.7962	0.2382	Up
PC aa C32:2	0.002473	2.607	0.8051	0.2514	Up
SM (OH) C24:1	0.00465	2.333	0.8101	0.2723	Up
PC ae C42:4	0.003172	2.499	0.8373	0.2689	Up
PC ae C30:2	0.000458	3.339	0.8534	0.2261	Up
PC ae C38:4	0.000229	3.641	0.8723	0.2181	Up
PC aa C28:1	0.000184	3.736	0.8887	0.2184	Up
PC aa C42:6	0.001126	2.949	0.9307	0.2678	Up
PC ae C34:3	0.000162	3.791	0.9337	0.2273	Up
PC ae C38:3	0.00006	4.225	1.024	0.2317	Up

## Discussion

This preliminary study showed a distinct lipidomic profile in the serum of fearful dogs, with increased concentrations of PCs compared to other lipid classes. In particular, our results describe changes within the circulating lipid pool of the serum compartment. PCs constitute about 50% of the total phospholipid mass of cellular membranes and their organelles [9], but direct extrapolations from circulating profiles to central nervous system structural lipids should be viewed as speculative hypotheses. Nevertheless, the statistical robustness of this intra-cohort comparison remains, and the distinct serum lipidomic fingerprints of fearful dogs and controls point to a clear metabolic divergence related to the behavioral phenotype. The prevalence of medium- and long-chain polyunsaturated PCs suggests involvement of systemic lipid transport pathways, which might secondarily correlate with synaptic membrane remodeling. Several biological processes might account for the observed increase of PC levels in this study. First, this lipidomic pattern may reflect reduced hepatic clearance or altered oxygen utilization, consistent with the oxidative stress previously reported in fearful dogs [13]. Moreover, changes in circulating PCs could partially result from alterations in hepatic metabolism driven by intestinal dysbiosis, as suggested by previous studies [16,31]. The correlation between lipid profiles and gut microbial composition may reveal common pathways (e.g., choline metabolism and neurotransmitter synthesis) linking emotional state, intestinal function, and systemic metabolic regulation [16]. An additional hypothesis not directly attained from serum data would correlate the accumulation of circulating PCs with a peripheral compensatory response to changes in neuronal membrane fluidity, involving the mobilization of systemic lipids to support synaptic remodeling. Finally, any interpretation of PC alterations in the context of membrane dynamics should also consider cholesterol, which co-regulates membrane fluidity, domain formation, and receptor activity alongside polyunsaturated phospholipids [32,33]. Translational evidence suggests that systemic lipid dysregulation may correlate with central mechanisms through shared metabolic pathways. Studies in mouse models of anxiety and in humans with depression have reported altered PC profiles, although the specific patterns vary across studies and biological contexts [10,34,35]. Specifically, neurotoxic agent-exposed mice exhibit anxiety-like behaviors and abnormally high concentrations of ether-linked PCs [36]; furthermore, elevated levels of glycerophospholipids, including 33 out of 35 PC species, were documented in AX inbred mouse strain exhibiting pronounced anxiety-like behaviors [10]. Similarly, elevated PC levels in the bloodstream of individuals with major depressive disorder align with our findings and support their potential as diagnostic biomarkers [35,37]. The functional meaning of these alterations remains debated: phospholipids are essential components of neuronal membranes and their metabolism is implicated in the pathophysiology of affective disorders [12,14]. However, it remains unclear whether elevated serum PC levels represent a causal factor, a secondary consequence of altered neuronal membrane dynamics or a compensatory response to chronic stress [12,14]. These cross-species observations support the hypothesis that systemic lipid dysregulation represents a common hallmark of affective dysfunction. However, methodological considerations must be acknowledged when comparing across studies; multiple factors, including the biological matrix, species, and disease stage, influence the direction of lipid changes. In particular, serum and plasma are distinct blood preparations with unique pre-analytical characteristics [38], and comparisons across species involve different matrices and disease stages. Lipidome data in dogs is limited and may appear inconsistent across studies. Specifically, our findings differ from those presented by Puurunen et al. (2016), who reported reduced phospholipids in fearful Great Danes. These apparently divergent molecular profiles likely reflect both technical factors and biological heterogeneity between cohorts. Of note, this study was performed on serum samples, while the above research used whole blood. The choice of matrix, whether including anticoagulants during sample collection, can significantly affect the detectable lipid profile due to enzymatic and metabolic processes occurring during blood coagulation [39,40]. Moreover, lipid profiles are sensitive to the specific extraction procedures, with substantial recovery biases for different lipid classes depending on the protocols employed [41,42]. Such technical differences are likely to contribute to the divergent molecular signatures observed between canine cohorts. Beyond analytical factors, biological variability also plays a significant role. The population of Puurunen et al. was composed of purebred Great Danes, whereas our cohort comprised mixed-breed dogs, more heterogeneous for genetic background and stress susceptibility. Moreover, mean age and body weight differed significantly between studies, factors known to influence lipid metabolism [43,44]. Furthermore, the severity and duration of fearful symptoms were not directly comparable between studies, and the present cohort included both males and females without sex-specific stratification, thus increasing demographic variability. Despite these differences, the observed changes remain statistically significant in our context, supporting the discriminatory potential of peripheral lipidomic profiles. From a clinical perspective, the identified lipid profile could provide support for differentiating fear disorders in dogs, which currently relies solely on subjective behavioral assessments. The high AUC value of 0.949 demonstrates good discriminatory power; however, further validation in larger, independent cohorts is required prior to clinical application. If confirmed, these lipid biomarkers could support early diagnosis in animals with ambiguous clinical presentation, monitor response to therapeutic interventions (pharmacological or behavioral), and identify patient subgroups with different biological profiles potentially responsive to targeted therapeutic strategies (e.g., supplementation with lipid precursors or membrane modulators). In conclusion, this preliminary study suggests the existence of a distinctive serum lipidomic signature associated with fear-related behaviours in dogs, characterised by elevated concentrations of PCs. Although the causal direction of this association remains to be determined, the differential lipidomic profile observed between the two cohorts supports the potential of peripheral lipid profiling as a complementary tool in the objective characterisation of canine neurobehavioral disorders. Future investigations should ideally combine lipidomics with complementary omic layers, including metabolomics, transcriptomics, and microbiome profiling, to identify coordinated alterations between circulating lipid metabolism, hepatic pathways, and gut microbial activity. Longitudinal studies, including evaluation of dogs before and after exposure to stressful conditions or behavioral interventions, would be particularly informative to determine whether the observed lipidomic changes represent stable metabolic traits or transient responses associated with emotional state.

## Limitations

We acknowledge that the small sample size (n = 8 per group) and demographic differences (particularly in the distribution by sex and body weight) between the groups limit the generalizability of our findings. The over-representation of small-sized spayed females in the fearful cohort corresponds to the demographic distribution reported in epidemiological studies on fear in dogs [45], although we acknowledge that our specific sample size may exhibit a referral bias towards our behavioral medicine service. In addition, the absence of free cholesterol data in this study precludes definitive conclusions on this aspect, and the observed PC alterations cannot be fully contextualized within the broader framework of membrane lipid homeostasis. Additional studies using larger, matched cohorts, considering biological matrix of sampling, sex, breed size, and reproductive status, are needed to confirm these preliminary findings.

## Abbreviations

AUC: Area Under the Curve
CAD: Collision gas
CUR: Curtain gas
ESI: Electrospray Ionization
FIA–MS/MS: Flow Injection Analysis–Tandem Mass Spectrometry
GS1/2: Ion source gas 1/2
IS: Ion Spray
MRM: Multiple Reaction Monitoring
MSEA: Metabolite Set Enrichment Analysis
NaN: Not a Number
PBS: Phosphate Buffered Saline
PC aa: Diacyl Phosphatidylcholines
PC ae: Acyl-Alkyl Phosphatidylcholines
PLS-DA: Partial Least Squares Discriminant Analysis
QC: Quality Control
ROC: Receiver Operating Characteristic
SM: Sphingomyelins
TG: Triacylglycerols
VIP: Variable Importance in Projection.
