# Supplementary material for: Targeted lipidomics showed altered serum phosphatidylcholine levels in fearful dogs
Source: PLoS One. 2026 Sep 2;21(9):e0356063. doi: 10.1371/journal.pone.0356063 (PMC13537564; doi:10.1371/journal.pone.0356063)
Supplement: Peer review history

# Peer review: Decision Letter 0

**DOI:** 10.1371/journal.pone.0356063.r001

**Authors:** Anil Bhatia

6 May 2026

Dear Dr. Napolitano,

Thank you for submitting your manuscript to PLOS ONE. After careful consideration, we feel that it has merit but does not fully meet PLOS ONE’s publication criteria as it currently stands. Therefore, we invite you to submit a revised version of the manuscript that addresses the points raised during the review process.

Please submit your revised manuscript by Jun 20 2026 11:59PM. If you will need more time than this to complete your revisions, please reply to this message or contact the journal office at plosone@plos.org. When you're ready to submit your revision, log on to https://www.editorialmanager.com/pone/ and select the 'Submissions Needing Revision' folder to locate your manuscript file.

- A letter that responds to each point raised by the academic editor and reviewer(s). You should upload this letter as a separate file labeled 'Response to Reviewers'.
- A marked-up copy of your manuscript that highlights changes made to the original version. You should upload this as a separate file labeled 'Revised Manuscript with Track Changes'.
- An unmarked version of your revised paper without tracked changes. You should upload this as a separate file labeled 'Manuscript'.

If you would like to make changes to your financial disclosure, please include your updated statement in your cover letter. Guidelines for resubmitting your figure files are available below the reviewer comments at the end of this letter.

If applicable, we recommend that you deposit your laboratory protocols in protocols.io to enhance the reproducibility of your results. Protocols.io assigns your protocol its own identifier (DOI) so that it can be cited independently in the future. For instructions see: https://journals.plos.org/plosone/s/submission-guidelines#loc-laboratory-protocols. Additionally, PLOS ONE offers an option for publishing peer-reviewed Lab Protocol articles, which describe protocols hosted on protocols.io. Read more information on sharing protocols at https://plos.org/protocols?utm_medium=editorial-email&utm_source=authorletters&utm_campaign=protocols.

As the corresponding author, your ORCID iD is verified in the submission system and will appear in the published article. PLOS supports the use of ORCID, and we encourage all coauthors to register for an ORCID iD and use it as well. Please encourage your coauthors to verify their ORCID iD within the submission system before final acceptance, as unverified ORCID iDs will not appear in the published article. *Only*  the individual author can complete the verification step; PLOS staff *cannot*  verify ORCID iDs on behalf of authors.

We look forward to receiving your revised manuscript.

Kind regards,

Anil Bhatia, Ph.D

Academic Editor

PLOS One

Journal Requirements:

When submitting your revision, we need you to address these additional requirements.

1.Please ensure that your manuscript meets PLOS ONE's style requirements, including those for file naming. The PLOS ONE style templates can be found at

https://journals.plos.org/plosone/s/file?id=wjVg/PLOSOne_formatting_sample_main_body.pdf and

https://journals.plos.org/plosone/s/file?id=ba62/PLOSOne_formatting_sample_title_authors_affiliations.pdf

2. If the reviewer comments include a recommendation to cite specific previously published works, please review and evaluate these publications to determine whether they are relevant and should be cited. There is no requirement to cite these works unless the editor has indicated otherwise.

[Note: HTML markup is below. Please do not edit.]

Reviewers' comments:

Reviewer's Responses to Questions

**Comments to the Author**

1. Is the manuscript technically sound, and do the data support the conclusions?

Reviewer #1: Partly

Reviewer #2: Partly

**********

2. Has the statistical analysis been performed appropriately and rigorously?

Reviewer #1: Yes

Reviewer #2: Yes

**********

3. Have the authors made all data underlying the findings in their manuscript fully available?

The PLOS Data policy

Reviewer #1: No

Reviewer #2: Yes

**********

4. Is the manuscript presented in an intelligible fashion and written in standard English?

Reviewer #1: Yes

Reviewer #2: Yes

**********

Reviewer #1: This is an interesting study, and it is encouraging to see the use of commercially available kits and established methodologies for mass spectrometry-based sample preparation and analysis. I have several questions and suggestions which, if addressed, would help clarify key aspects of the study and strengthen its overall rigor:

- In the Introduction, please elaborate on the importance of quantifiable biomarkers in canines. Specifically, it would be helpful to clarify why behavioral analyses alone may be insufficient, supported by appropriate references.

- The manuscript notes that subjects were withdrawn from therapy and psychotropic medications for four weeks. Please provide supporting literature demonstrating that this duration is sufficient for behavioral, fear, or anxiety responses to return to baseline levels.

- In this context, consider including a supplemental table detailing each subject’s prior psychotropic medications (including dosage) as well as the types of therapy they were receiving.

- In the mass spectrometry Methods section, please include full source and instrument parameters for the triple quadrupole (e.g., temperature, gas flows/pressures, voltages). Additionally, a supplemental table outlining the monitored MRM transitions would be valuable.

- The figures are currently of low resolution; please provide higher-quality versions to improve clarity.

- The Conclusion references potential multi-omic approaches. Please expand on this point by outlining the types of studies or experimental designs that would be required to substantiate or refute the claims suggested by this preliminary work.

Reviewer #2: The work by Sacchettino and colleagues entitled “Targeted lipidomics showed altered serum phosphatidylcholine levels in fearful dogs: a preliminary study” describes the detection and quantification of 523 lipid species by targeted MS approaches in serum samples collected from fearful dogs (n=8) and compared against healthy dogs (n=8) showing spontaneous interactions and no behavioral manifestations (fear, anxiety). Lipidomic statistical analysis by available online platform MetaboAnalyst 6.0, results reveal a panel of 26, out of 523 lipid species, differentially elevated in case group (fearful dogs) compared to control group leading the authors to conclude that dysregulation of PC lipids could potentially be linked to canine behavioral disorders. While the authors identify the limitations of the current study, results from this exploratory study underpins the potential of lipidomic studies to complement clinical evaluation and improve decision-making in veterinary environments.

The work by Sacchettino and colleagues is another example of the potential value of circulating lipid fingerprinting could improve the decision-making during the onset, progression and management of diseases. However, their discussion of results is somewhat dense without a clear and straightforward contextualization of findings (please see below) that must be improved for clarity. Based on this, the work cannot be accepted in its present form.

Major points:

1. Introduction.

• While behavioral disorders are linked to alterations in brain chemistry, structure and function, the link between these changes and the blood signatures is not obvious to the reader, and the reasons why the authors opted for serum lipid analysis and not cerebral spinal fluid metabolite analysis in the search for objective and quantifiable biomarkers to complement behavioral evaluation.

2. Material & Methods

• The authors state that 523 lipid species were detected and quantified in canine sera samples and that “…the features with high percentage (>50%) of missing values or non-valid values were discarded in the post-analysis” (lines 131-133). How many of the 523 quantified lipids were discarded during this processing stage? This needs to be included and clarified in the Results Sections.

• According to the authors, lipid extracts were prepared using 5 mM ammonium acetate in methanol. The authors need to include additional details on the volumes used. How much volume of serum? How much volume of methanol with 5mM ammonium acetate was used in the extraction step? As well as speed and time of centrifugation. The goal of including experimental details is so that others keen to investigate this topic may use similar conditions that in turn be used for comparison purposes.

• Considering that extraction of lipid from aqueous biological matrices is typically carried out using popular extraction protocols (Folch, Bligh & Dyer, Matyash, ACN precipitation) which have been extensively studied (please see works by Matyash et al., J Lipid Res (2008) 49(5):1137-46. doi: 10.1194/jlr.D700041-JLR200; Reis et al., J Lipid Res. (2013) 54(7):1812–1824. doi: 10.1194/jlr.M034330; Sarafian et al., Anal Chem (2014) 86(12):5766-74. doi: 10.1021/ac500317c) please clarify the reason behind the preference of 5 mM ammonium acetate in methanol. What is the extraction performance of this solvent in the extraction of lipids (for the predominant and less abundant lipid classes)?

• Also, in line 141 the authors state “…10 μL of samples were diluted with 490 μL of FIA solvent”. Surely, they mean 10 μL of lipid extract not serum sample. Please clarify.

3. Results

• PC lipid species quantified and shown in Figure 2 reveal concentration values ranging from sub-micromolar as shown for PC ae C30:2 (0.0-0.4 �M) to millimolar values as shown for PC aa C38:4 (0-2.5mM). Given the wide range of concentration values reported for abundant and residual lipids in serum samples analyzed, the authors need to include information about method validation parameters, including linearity, LOD and LOQ obtained for the various calibration curves (standards). This can be included in Supplementary Information Section. If this was done elsewhere and is previously published, the authors need to include the information in the Methods Section or in alternative the reference of the work.

4. Discussion.

• In their attempt to contextualize results with the published literature, the authors interchangeably shift the focus of their discussion from circulating lipids to membrane lipids (lines 251-259, lines 265-266), compare their serum results with plasma and tissue samples (lines 282-283, lines 303-310) obtained from dogs with mouse, mice and humans. This is confusing to the readers, particularly the non-expert audiences keen to learn about this topic. The overall discussion is not coherent and straightforward as in the end, the discrepancies of findings between this and other studies may be mostly attributed to the inclusion of different matrices (plasma vs serum vs tissue) from different species (dogs, mice, mouse, humans), with different disorders (some medicated) and at different stages of the disease, parameters that affect the data and hence not comparable. Comparison of lipidomic data obtained through different sample collection (with or no anti-coagulant agents), with different lipid extraction protocols (one-phase and two-phase protocols, with hexane, MeOH or chlorinated organic solvents (dichloromethane or chloroform), and under medication are factors that alter the lipid fingerprint in biological samples (please see works by Cruickshank-Quin et al., Metabolites (2018) 8(4):88. doi: 10.3390/metabo8040088; Ishikawa et al., PLoS One. (2014) 9(3):e91806. doi: 10.1371/journal.pone.0091806; Reis et al., J Lipid Res. (2013) 54(7):1812–1824. doi: 10.1194/jlr.M034330; Croyal et al., Diabetes Metab (2018) 44(2):143-149. doi: 10.1016/j.diabet.2017.04.003). Moreover, as acknowledged by the authors themselves, the heterogeneity of samples included in this study (case and control groups) is very broad due to low sampling (n=8), with groups comprised of mixed-breed dogs, gender-biased, with different age and weight, severity and duration of symptoms (lines 287-288; line 295, Table 2). The authors are advised to take all these aspects into consideration when restructuring their discussion.

• Likewise, the comparison of serum data from this study with results obtained from plasma (line 308), or even and whole blood samples (line 282-283), is arguable as plasma and serum samples are very different matrices in terms of their metabolite composition. Previous studies have shown that because serum is susceptible to variations in clotting time and temperature, and analysis of plasma samples provides higher reproducibility for clinical metabolomics (please see work by Yu et al., PLSOne (2011) 6, e21230 doi: 10.1371/journal.pone.0021230; and Zubkowski et al., Metabolomics (2025) 21, doi:146 10.1007/s11306-025-02345-w).

• As acknowledged by the authors, alterations in circulating lipids reflect modifications in hepatic metabolism induced by intestinal dysbiosis (lines 327-328). Considering that lipids are transported in circulation by lipoproteins (macromolecular aggregates), it is not clear why the authors did not focus their characterization to HDL phospholipid-rich particles to test their hypothesis of better understand the correlation of lipids and behavioral disorders. This is even more relevant considering that HDL particles are the predominant carrier of lipids in circulation in canine blood samples (please see work by Kaabia et al., Sci Rep (2018) 8(1):15893. doi: 10.1038/s41598-018-34329-3). Please comment.

• As stated by the authors “Polyunsaturated PCs are essential constituents of neuronal membranes, where they affect membrane fluidity, the clustering of neurotransmitter receptors, and cellular signaling [30, 31]”. However, neuronal cells are also enriched in cholesterol (please see work by Fitzner et al., Cell Rep. (2020) 32(11):108132. doi: 10.1016/j.celrep.2020.108132) with massive implications to membrane’s fluidity, permeability, diffusion and signaling (please see work by Ingólfsson et al., Biophys J. (2017) 113(10):2271–2280. doi: 10.1016/j.bpj.2017.10.017). Though quantification of cholesterol was not conducted by the authors through FIA-MS/MS approach, its contribution to neuronal membrane biophysical and biological properties should be mentioned in the discussion section.

• In their statement “… it is worth mentioning that targeted lipidomics does not allow to outline an overall molecular signature, which represents one of the main drawbacks compared with the non-targeted approach [36]” (lines 293-295) the authors redirect the reader’s attention to a potential drawback of targeted approaches over untargeted approaches. Based on this reviewer’s experience in lipid analysis by targeted and untargeted approaches, the sentence is misleading to the non-expert audience, as lipid analysis by untargeted approaches, either by direct injection or coupled to chromatography, do not provide a “true” lipidomic signature (information on the lipid species and amounts) as ion intensities do not reflect lipid abundance but rather lipid ionization efficiencies. Lipid classes with higher ionization efficiency will lead to higher ion intensities in shotgun MS spectra or LC-MS chromatograms. A perfect example is lyso-phosphatidylcholines (LPC) that display high ion intensities (abundant chromatographic bands when compared to cholesteryl esters) due to higher ionization efficiencies but generally present in lower concentration values. This is particularly relevant as liquid chromatography (LC) were not coupled to MS instruments which could have minimize ion suppression effects.

5. References

• The majority of references (47%) are over 5 years old, which is understandable considering the low number of works focusing on dog circulating lipidome.

• Also, 8 out of 42 references (19%) are self-citations, corresponding to nearly one-fifth of the total citations, which seem excessive.

Other points

• The authors state that “Samples of the main groups (case and control) that deviated significantly from the main data cluster upon PLS-DA were recognized as outliers and excluded from the data set.” (lines 161-162). Surely the authors mean replicates not samples (bearing in mind sentence in lines 153-154). Considering that the study contains a poor sampling (n=8), exclusion of samples (in triplicates) will reduce even more the number of points in the dataset. Please clarify.

• Figures 1 and 2 show poor resolution.

• Several abbreviations are not obvious to the reader. What does NaN stand for (line 150)? The inclusion of a list of abbreviations would be helpful.

**********

what does this mean?). If published, this will include your full peer review and any attached files.

If you choose “no”, your identity will remain anonymous but your review may still be made public.

**Do you want your identity to be public for this peer review?**  For information about this choice, including consent withdrawal, please see our Privacy Policy

Reviewer #1: No

Reviewer #2: **Yes:** Ana Reis

**********

[NOTE: If reviewer comments were submitted as an attachment file, they will be attached to this email and accessible via the submission site. Please log into your account, locate the manuscript record, and check for the action link "View Attachments". If this link does not appear, there are no attachment files.]

To ensure your figures meet our technical requirements, please review our figure guidelines: https://journals.plos.org/plosone/s/figures

You may also use PLOS’s free figure tool, NAAS, to help you prepare publication quality figures: https://journals.plos.org/plosone/s/figures#loc-tools-for-figure-preparation.

NAAS will assess whether your figures meet our technical requirements by comparing each figure against our figure specifications.

# Author comment: Author response to Decision Letter 1

**DOI:** 10.1371/journal.pone.0356063.r002

1 Jul 2026

Point-by-point response to reviewers’ comments

Reviewer #1:

This is an interesting study, and it is encouraging to see the use of commercially available kits and established methodologies for mass spectrometry-based sample preparation and analysis. I have several questions and suggestions which, if addressed, would help clarify key aspects of the study and strengthen its overall rigor:

Q. In the Introduction, please elaborate on the importance of quantifiable biomarkers in canines. Specifically, it would be helpful to clarify why behavioral analyses alone may be insufficient, supported by appropriate references.

A. We agree with the Reviewer that justifying the shift from purely behavioral to molecular assessment is crucial for the manuscript. We have expanded the Introduction to discuss the inherent subjectivity of behavioral observation and owner reports, citing the potential for observer bias and the inability of behavioral signs alone to distinguish between different underlying physiological states. We hope that the revised text now emphasizes how quantifiable biomarkers are essential for an evidence-based approach in veterinary behavioral medicine, aiding in more precise diagnosis and monitoring of therapeutic efficacy.

Q. The manuscript notes that subjects were withdrawn from therapy and psychotropic medications for four weeks. Please provide supporting literature demonstrating that this duration is sufficient for behavioral, fear, or anxiety responses to return to baseline levels.

A. We thank the Reviewer for the observation. There was a misunderstanding in the manuscript description. The animals included in the fearful group were treatment-naive. No dogs had ever received psychotropic medications, supplements, or behavioral therapy prior to the study. The mention of four weeks was intended as a minimum safety interval to exclude any recent occasional administration, not as a wash-out period from chronic therapy.

Q. In this context, consider including a supplemental table detailing each subject’s prior psychotropic medications (including dosage) as well as the types of therapy they were receiving.

A. Since no previous treatments occurred, the behavioral and lipidomic responses represent the subjects' baseline levels. We have revised the manuscript to clarify this point.

Q. In the mass spectrometry Methods section, please include full source and instrument parameters for the triple quadrupole (e.g., temperature, gas flows/pressures, voltages). Additionally, a supplemental table outlining the monitored MRM transitions would be valuable.

A. Thank you for the suggestion. We have included the MS parameters in the Material and Methods section. With regards to the MRM transitions, according to the Biocrates manufacturer policy, they are not disclosed so we do not have any chance to publish them.

Q. The figures are currently of low resolution; please provide higher-quality versions to improve clarity.

A. We thank the reviewer. We included high-resolution figures in the new revised article.

Q. The Conclusion references potential multi-omic approaches. Please expand on this point by outlining the types of studies or experimental designs that would be required to substantiate or refute the claims suggested by this preliminary work.

A. We thank the reviewer for this valuable suggestion. We agree that the potential mechanisms proposed in our study require further validation through integrative multi-omic approaches. In the revised manuscript, we have expanded the Conclusion to better clarify the types of studies needed to complement or validate these preliminary findings.

Reviewer #2:

The work by Sacchettino and colleagues entitled "Targeted lipidomics showed altered serum phosphatidylcholine levels in fearful dogs: a preliminary study" describes the detection and quantification of 523 lipid species by targeted MS approaches in serum samples collected from fearful dogs (n=8) and compared against healthy dogs (n=8) showing spontaneous interactions and no behavioral manifestations (fear, anxiety). Lipidomic statistical analysis by available online platform MetaboAnalyst 6.0, results reveal a panel of 26, out of 523 lipid species, differentially elevated in case group (fearful dogs) compared to control group leading the authors to conclude that dysregulation of PC lipids could potentially be linked to canine behavioral disorders. While the authors identify the limitations of the current study, results from this exploratory study underpins the potential of lipidomic studies to complement clinical evaluation and improve decision-making in veterinary environments.

The work by Sacchettino and colleagues is another example of the potential value of circulating lipid fingerprinting could improve the decision-making during the onset, progression and management of diseases. However, their discussion of results is somewhat dense without a clear and straightforward contextualization of findings (please see below) that must be improved for clarity. Based on this, the work cannot be accepted in its present form.

Major points:

1. Introduction.

Q. While behavioral disorders are linked to alterations in brain chemistry, structure and function, the link between these changes and the blood signatures is not obvious to the reader, and the reasons why the authors opted for serum lipid analysis and not cerebral spinal fluid metabolite analysis in the search for objective and quantifiable biomarkers to complement behavioral evaluation.

A. We appreciate the Reviewer for highlighting this important issue, as it allows us to clarify the clinical and ethical logic of our experimental design. Cerebrospinal fluid (CSF) collection is not a conventional procedure for behavioral assessment in veterinary medicine. CSF sampling is an invasive procedure that generally requires anesthesia and carries clinical risks. Conversely, our study sought to identify readily accessible and minimally invasive peripheral biomarkers pertinent to practical veterinary applications. To achieve this goal, we used serum obtained from venous blood during routine annual health screenings, thus minimizing any additional stress or discomfort for the animals. We agree that a more detailed analysis of the pathophysiological relationship between peripheral blood signatures and central mechanisms is needed. The Introduction has been modified to clarify the translational significance of serum lipidomics and to justify our choice of matrix based on clinical feasibility and ethical considerations in companion animals.

2. Material & Methods

Q. The authors state that 523 lipid species were detected and quantified in canine sera samples and that “…the features with high percentage (>50%) of missing values or non-valid values were discarded in the post-analysis” (lines 131-133). How many of the 523 quantified lipids were discarded during this processing stage? This needs to be included and clarified in the Results Sections.

A. We thank the reviewer for this suggestion. We have specified in the Results the percentage of missing values in the total dataset. In addition, we have better explained that one lipid was discarded from our list as non-valid values were present in more than the 50% of the samples. Finally, the lipid dataset included 522 lipids.

Q. According to the authors, lipid extracts were prepared using 5 mM ammonium acetate in methanol. The authors need to include additional details on the volumes used. How much volume of serum? How much volume of methanol with 5mM ammonium acetate was used in the extraction step? As well as speed and time of centrifugation. The goal of including experimental details is so that others keen to investigate this topic may use similar conditions that in turn be used for comparison purposes.

A. We thank the reviewer for this suggestion. We have expanded the Materials and Methods section regarding lipidomic sample preparation with the aim to including all the procedural details.

Q. Considering that extraction of lipid from aqueous biological matrices is typically carried out using popular extraction protocols (Folch, Bligh & Dyer, Matyash, ACN precipitation) which have been extensively studied (please see works by Matyash et al., J Lipid Res (2008) 49(5):1137-46. doi: 10.1194/jlr.D700041-JLR200; Reis et al., J Lipid Res. (2013) 54(7):1812–1824. doi: 10.1194/jlr.M034330; Sarafian et al., Anal Chem (2014) 86(12):5766-74. doi: 10.1021/ac500317c) please clarify the reason behind the preference of 5 mM ammonium acetate in methanol. What is the extraction performance of this solvent in the extraction of lipids (for the predominant and less abundant lipid classes)?

A. We thank the reviewer for this important comment. The use of 5 mM ammonium acetate in methanol was selected according to the standardized extraction protocol provided by Biocrates for the MxP Quant 500 platform and was not intended as an alternative to classical lipid extraction procedures (Folch, Bligh & Dyer, Matyash, or ACN precipitation) that may provide optimal recovery for comprehensive lipidomic profiling.

The main rationale for this extraction approach is that the Biocrates workflow is specifically designed to enable the parallel extraction and quantification of a broad range of analytes from a single biological sample: small metabolites (amino acids, amino acid related, bile acids, carboxylic acids etc.) that are run by LC-MS/MS and various lipid classes (acylcarnitines, glycerophospholipids, sphingolipids, etc.) that are run by FIA-MS/MS. For this study, we focused solely on the lipid panel.

While dedicated lipid extraction protocols may provide higher recovery for specific lipid subclasses, the targeted approach validated by Biocrates may represents a good compromise between extraction efficiency across chemically diverse metabolite classes and analytical reproducibility, providing throughput comparable to untargeted methods but with higher accuracy and sensitivity.

Q. Also, in line 141 the authors state “…10 μL of samples were diluted with 490 μL of FIA solvent”. Surely, they mean 10 μL of lipid extract not serum sample. Please clarify.

A. This is completely right, we meant “10 μL of lipid extract”. We have better explained this in the main text.

3. Results

Q. PC lipid species quantified and shown in Figure 2 reveal concentration values ranging from sub-micromolar as shown for PC ae C30:2 (0.0-0.4 µM) to millimolar values as shown for PC aa C38:4 (0-2.5mM). Given the wide range of concentration values reported for abundant and residual lipids in serum samples analyzed, the authors need to include information about method validation parameters, including linearity, LOD and LOQ obtained for the various calibration curves (standards). This can be included in Supplementary Information Section. If this was done elsewhere and is previously published, the authors need to include the information in the Methods Section or in alternative the reference of the work.

The MxP® Quant 500 kit is a fully validated targeted metabolomics platform. Quantification of FIA-MS/MS analytes is based on isotopically labelled internal standards and kit-specific calibration procedures, but not on individual calibration curves for each analyte. Analytical performance characteristics, including linearity, LOD, LLOQ, ULOQ, accuracy and precision, have been extensively validated by Biocrates and are provided in the kit analytical validation documentation.

A.We have better explained the quantification strategy in the Methods section and have included corresponding references.

4. Discussion.

Q. In their attempt to contextualize results with the published literature, the authors interchangeably shift the focus of their discussion from circulating lipids to membrane lipids (lines 251-259, lines 265-266), compare their serum results with plasma and tissue samples (lines 282-283, lines 303-310) obtained from dogs with mouse, mice and humans. This is confusing to the readers, particularly the non-expert audiences keen to learn about this topic. The overall discussion is not coherent and straightforward as in the end, the discrepancies of findings between this and other studies may be mostly attributed to the inclusion of different matrices (plasma vs serum vs tissue) from different species (dogs, mice, mouse, humans), with different disorders (some medicated) and at different stages of the disease, parameters that affect the data and hence not comparable. Comparison of lipidomic data obtained through different sample collection (with or no anti-coagulant agents), with different lipid extraction protocols (one-phase and two-phase protocols, with hexane, MeOH or chlorinated organic solvents (dichloromethane or chloroform), and under medication are factors that alter the lipid fingerprint in biological samples (please see works by Cruickshank-Quin et al., Metabolites (2018) 8(4):88. doi: 10.3390/metabo8040088; Ishikawa et al., PLoS One. (2014) 9(3):e91806. doi: 10.1371/journal.pone.0091806; Reis et al., J Lipid Res. (2013) 54(7):1812–1824. doi: 10.1194/jlr.M034330; Croyal et al., Diabetes Metab (2018) 44(2):143-149. doi: 10.1016/j.diabet.2017.04.003). Moreover, as acknowledged by the authors themselves, the heterogeneity of samples included in this study (case and control groups) is very broad due to low sampling (n=8), with groups comprised of mixed-breed dogs, gender-biased, with different age and weight, severity and duration of symptoms (lines 287-288; line 295, Table 2). The authors are advised to take all these aspects into consideration when restructuring their discussion.

A. We understand the Reviewer’s point regarding the impact of pre-analytical and methodological variables. It is scientifically true that differences in biological matrices, sample collection protocols (with or without anticoagulants), and chemical extraction methods directly affect the lipid fingerprint, making direct quantitative comparisons difficult. We have addressed the issue by restructuring the Discussion to clarify these technical discrepancies. However, we want to emphasize that the primary objective and focus of our study was the intra-species comparison between the serum of dogs with generalized fear and normo-behavioral controls within our own controlled, treatment-naive cohort. Despite the limited sample size, the serum lipidomic fingerprints of these two groups are strongly distinct and statistically different. This significant variance constitutes an intriguing, preliminary finding that warrants further investigation. As the Reviewer rightly notes in a subsequent comment, the published literature on canine behavioral lipidomics is extremely scarce. Because of this severe lack of data, referencing external studies—even those involving different species, matrices, or disease stages—was a necessary approach to contextualize our preliminary findings within a broader biological framework. We acknowledged these cross-species and cross-matrix differences as limitations in our original text to maintain scientific transparency. In conclusion, while we agree that our study is preliminary and requires validation in a larger, more homogenous cohort, our current data remain statistically robust and offer a novel perspective in a field where literature is lacking. We have refined the text to clarify these points, ensuring a sharp distinction between our solid, statistically significant intra-cohort findings and the broader, cross-species literature used for contextual discussion.

Q. Likewise, the comparison of serum data from this study with results obtained from plasma (line 308), or even and whole blood samples (line 282-283), is arguable as plasma and serum samples are very different matrices in terms of their metabolite composition. Previous studies have shown that because serum is susceptible to variations in clotting time and temperature, and analysis of plasma samples provides higher reproducibility for clinical metabolomics (please see work by Yu et al., PLSOne (2011) 6, e21230 doi: 10.1371/journal.pone.0021230; and Zubkowski et al., Metabolomics (2025) 21, doi:146 10.1007/s11306-025-02345-w).

A. We would like to thank the Reviewer for her comments. We have restructured the discussion to address the differences

Attachment Submitted filename: Sacchettino et al. 2026-Response to Reviewers letter.docx

# Peer review: Decision Letter 1

**DOI:** 10.1371/journal.pone.0356063.r003

**Authors:** Anil Bhatia

30 Jul 2026

Targeted lipidomics showed altered serum phosphatidylcholine levels in fearful dogs: a preliminary study

PONE-D-26-16475R1

Dear Dr. Napolitano,

We’re pleased to inform you that your manuscript has been judged scientifically suitable for publication and will be formally accepted for publication once it meets all outstanding technical requirements.

Within one week, you’ll receive an e-mail detailing the required amendments. When these have been addressed, you’ll receive a formal acceptance letter and your manuscript will be scheduled for publication.

An invoice will be generated when your article is formally accepted. Please note, if your institution has a publishing partnership with PLOS and your article meets the relevant criteria, all or part of your publication costs will be covered. Please make sure your user information is up-to-date by logging into Editorial Manager at Editorial Manager® and clicking the ‘Update My Information' link at the top of the page. For questions related to billing, please contact billing support.

If your institution or institutions have a press office, please notify them about your upcoming paper to help maximize its impact. If they’ll be preparing press materials, please inform our press team as soon as possible -- no later than 48 hours after receiving the formal acceptance. Your manuscript will remain under strict press embargo until 2 pm Eastern Time on the date of publication. For more information, please contact onepress@plos.org.

Kind regards,

Anil Bhatia, Ph.D

Academic Editor

PLOS One

Additional Editor Comments (optional):

Reviewers' comments:

Reviewer's Responses to Questions

**Comments to the Author**

Reviewer #1: All comments have been addressed

Reviewer #2: All comments have been addressed

**********

2. Is the manuscript technically sound, and do the data support the conclusions?

Reviewer #1: Yes

Reviewer #2: Yes

**********

3. Has the statistical analysis been performed appropriately and rigorously?

Reviewer #1: Yes

Reviewer #2: Yes

**********

4. Have the authors made all data underlying the findings in their manuscript fully available?

The PLOS Data policy

Reviewer #1: Yes

Reviewer #2: Yes

**********

5. Is the manuscript presented in an intelligible fashion and written in standard English?

Reviewer #1: Yes

Reviewer #2: Yes

**********

Reviewer #1: The authors have adequately addressed the comments raised during the previous round of review. The revisions have improved the clarity and comprehensiveness of the manuscript, and the added discussion appropriately acknowledges and addresses the study's limitations. Overall, the manuscript is now well presented, and I believe it is suitable for publication in its current form. I recommend acceptance.

Reviewer #2: The autors have addressed the reviewer's comments.

Figures still have poor resolution though this may something concerning the picture format and should be pursued and addressed during the final proofreading stages.

**********

what does this mean?). If published, this will include your full peer review and any attached files.

If you choose “no”, your identity will remain anonymous but your review may still be made public.

**Do you want your identity to be public for this peer review?**  For information about this choice, including consent withdrawal, please see our Privacy Policy

Reviewer #1: No

Reviewer #2: No

**********

# Editor report: Acceptance letter

**DOI:** 10.1371/journal.pone.0356063.r004

**Authors:** Anil Bhatia

PONE-D-26-16475R1

PLOS One

Dear Dr. Napolitano,

I'm pleased to inform you that your manuscript has been deemed suitable for publication in PLOS One. Congratulations! Your manuscript is now being handed over to our production team.

At this stage, our production department will prepare your paper for publication. This includes ensuring the following:

* All references, tables, and figures are properly cited

* All relevant supporting information is included in the manuscript submission,

* There are no issues that prevent the paper from being properly typeset

You will receive further instructions from the production team, including instructions on how to review your proof when it is ready. Please keep in mind that we are working through a large volume of accepted articles, so please give us a few days to review your paper and let you know the next and final steps.

Lastly, if your institution or institutions have a press office, please let them know about your upcoming paper now to help maximize its impact. If they'll be preparing press materials, please inform our press team within the next 48 hours. Your manuscript will remain under strict press embargo until 2 pm Eastern Time on the date of publication. For more information, please contact onepress@plos.org.

You will receive an invoice from PLOS for your publication fee after your manuscript has reached the completed accept phase. If you receive an email requesting payment before acceptance or for any other service, this may be a phishing scheme. Learn how to identify phishing emails and protect your accounts at https://explore.plos.org/phishing.

If we can help with anything else, please email us at customercare@plos.org.

Thank you for submitting your work to PLOS ONE and supporting open access.

Kind regards,

PLOS ONE Editorial Office Staff

on behalf of

Dr. Anil Bhatia

Academic Editor

PLOS One
